# Amplicon sequences of sourdough starter cultures treated with varying levels of water chlorination

**DOI:** 10.1128/mra.01001-24

**Published:** 2024-12-27

**Authors:** Pearson Lau, Swapan Jain, Rei Peraza, Gabriel G. Perron

**Affiliations:** 1 Department of Biology, Bard College, Annandale-On-Hudson, New York, USA; 2 Department of Chemistry, Bard College, Annandale-On-Hudson, New York, USA; 3 Rossi & Sons Alimentari, Poughkeepsie, New York, USA; 4 Center for Environmental Sciences and Humanities, Annandale-On-Hudson, New York, USA; 5 Center for Genomics and Systems Biology, New York University, New York, New York, USA; Queens College Department of Biology, Queens, New York, USA

**Keywords:** food fermentation, 16S amplicon, food microbiology, *Lactobacillus*

## Abstract

Here, we present amplicon sequences from sourdough starter cultures that have been treated with a chlorine concentration gradient mirroring public water distribution systems. Data derived present insights into the effect of important environmental factors that may influence the formation of microbial communities in food biomes.

## ANNOUNCEMENT

The quality of starting materials is a defining feature for the cultivation of specialized microbial communities in fermented food biomes, resulting in desired functional outputs (1). For example, flour source in sourdough making greatly influences the development of microbial communities (2). Water, being the other primary ingredient, is now appreciated as a driving force (3). However, understanding water quality itself as a driver has been an ongoing effort. Here, we present results of our amplicon sequencing of sourdough starters impacted by increasing water chlorination concentrations to address such question.

Methods are further described in our related work (4). Starters were generated under laboratory conditions at Bard College located in Annandale-on-Hudson, NY (42.0231° N, 73.9093° W) or prepared in a working kitchen environment (42.0590765–73.9100806). Briefly, sourdough starters were prepared using the traditional preparation method known as “backslopping.” Initial starters were mixed with equal proportions of flour (50 g) and water (50 mL) prepared with one of the following chlorine levels (4): 0, 0.5, and 4.0 mg/L. Backslopping was performed by removing 50% of the starter (50 g/mL), replenished with an equal ratio of flour (25 g):water (25 mL) to retain a consistent dough yield, and thoroughly mixed with the BagMixer 5000 (Interscience, Saint Nom la Brétèche, France). Starters were fermented under sterile laboratory air, where air temperature was maintained at 22°C–25°C, or unsterilized ambient air. Starters were manipulated under sterile conditions and sampled on days 1, 4, and 7.

Genomic DNA was obtained through diluting 1 g of the starter pre-backslopping to 9 mL 0.1% peptone and 0.85% NaCl solution to achieve a 10-fold dilution. One milliliter of the homogenized dilution was processed using the MoBio PowerFood DNA extraction kit (MoBio, Carlsbad, CA). Amplification of the V4 region of the *16S rRNA* gene was performed using primers 515F/806R to produce an approximate ~300 bp amplicon (5) and further gel purified before pooling at equimolar ratios. Pooled libraries were sequenced on an adapted MiSeq paired-end Illumina platform for 2 × 250 bp paired-end reads (Wright Labs, Huntingdon, PA) following the Earth Microbiome Project’s protocol (6). Detailed sample metadata are presented in Table 1.

**TABLE 1:** Summary metadata of sourdough starter samples^*a*^*^,^*^*b*^

ID	Label	Air	Day	Source	Chlorine	PPM	PPMf	*intI1*	pH
PL-1	Control_lab_1_1d	Filtered	1	Starter	Control	0	Control	NA	6.07
PL-2	Control_lab_2_1d	Filtered	1	Starter	Control	0	Control	NA	6.08
PL-3	Control_lab_3_1d	Filtered	1	Starter	Control	0	Control	NA	6.01
PL-4	Cl0.5_lab_1_1d	Filtered	1	Starter	Chlorine	0.5	Medium	NA	6.07
PL-5	Cl0.5_lab_2_1d	Filtered	1	Starter	Chlorine	0.5	Medium	NA	5.34
PL-6	Cl0.5_lab_3_1d	Filtered	1	Starter	Chlorine	0.5	Medium	NA	6.00
PL-7	Cl4_lab_1_1d	Filtered	1	Starter	Chlorine	4	High	NA	6.32
PL-8	Cl4_lab_2_1d	Filtered	1	Starter	Chlorine	4	High	NA	5.57
PL-9	Cl4_lab_3_1d	Filtered	1	Starter	Chlorine	4	High	NA	6.17
PL-10	Control_lab_4_1d	Filtered	1	Starter	Control	0	Control	NA	5.37
PL-11	Control_lab_5_1d	Filtered	1	Starter	Control	0	Control	NA	6.08
PL-12	Control_lab_6_1d	Filtered	1	Starter	Control	0	Control	NA	5.45
PL-13	Cl0.5_lab_4_1d	Filtered	1	Starter	Chlorine	0.5	Medium	NA	5.56
PL-14	Cl0.5_lab_5_1d	Filtered	1	Starter	Chlorine	0.5	Medium	NA	6.26
PL-15	Cl0.5_lab_6_1d	Filtered	1	Starter	Chlorine	0.5	Medium	NA	6.29
PL-16	Cl4_lab_4_1d	Filtered	1	Starter	Chlorine	4	High	NA	5.27
PL-17	Cl4_lab_5_1d	Filtered	1	Starter	Chlorine	4	High	NA	6.29
PL-18	Cl4_lab_6_1d	Filtered	1	Starter	Chlorine	4	High	NA	5.61
PL-19	Control_lab_1_7d	Filtered	7	Starter	Control	0	Control	NA	3.94
PL-20	Control_lab_2_7d	Filtered	7	Starter	Control	0	Control	NA	3.94
PL-21	Control_lab_3_7d	Filtered	7	Starter	Control	0	Control	NA	3.96
PL-22	Cl0.5_lab_1_7d	Filtered	7	Starter	Chlorine	0.5	Medium	NA	3.98
PL-23	Cl0.5_lab_2_7d	Filtered	7	Starter	Chlorine	0.5	Medium	NA	3.95
PL-24	Cl0.5_lab_3_7d	Filtered	7	Starter	Chlorine	0.5	Medium	NA	3.93
PL-25	Cl4_lab_1_7d	Filtered	7	Starter	Chlorine	4	High	NA	3.97
PL-26	Cl4_lab_2_7d	Filtered	7	Starter	Chlorine	4	High	NA	3.83
PL1	Control_1_4d	Unfiltered	4	Starter	Control	0	Control	2.80E-05	NA
PL2	Control_2_4d	Unfiltered	4	Starter	Control	0	Control	3.59E-05	NA
PL3	Control_3_4d	Unfiltered	4	Starter	Control	0	Control	1.43E-05	NA
PL4	Cl0.5_1_4d	Unfiltered	4	Starter	Chlorine	0.5	Medium	3.21E-05	NA
PL5	Cl0.5_2_4d	Unfiltered	4	Starter	Chlorine	0.5	Medium	0	NA
PL6	Cl0.5_3_4d	Unfiltered	4	Starter	Chlorine	0.5	Medium	0.000123	NA
PL7	Cl4_1_4d	Unfiltered	4	Starter	Chlorine	4	High	3.99E-05	NA
PL8	Cl4_2_4d	Unfiltered	4	Starter	Chlorine	4	High	0	NA
PL9	Cl4_3_4d	Unfiltered	4	Starter	Chlorine	4	High	0	NA
PL10	Control_1_7d	Unfiltered	7	Starter	Control	0	Control	0	NA
PL11	Control_2_7d	Unfiltered	7	Starter	Control	0	Control	9.46E-05	NA
PL12	Control_3_7d	Unfiltered	7	Starter	Control	0	Control	0.00041225	NA
PL13	Cl0.5_1_7d	Unfiltered	7	Starter	Chlorine	0.5	Medium	0.00016459	NA
PL14	Cl0.5_2_7d	Unfiltered	7	Starter	Chlorine	0.5	Medium	0.0003218	NA
PL15	Cl0.5_3_7d	Unfiltered	7	Starter	Chlorine	0.5	Medium	4.52E-05	NA
PL16	Cl4_1_7d	Unfiltered	7	Starter	Chlorine	4	High	0.00107936	NA
PL17	Cl4_2_7d	Unfiltered	7	Starter	Chlorine	4	High	0.00328553	NA
PL18	Cl4_3_7d	Unfiltered	7	Starter	Chlorine	4	High	0.00012288	NA
PL19	Flour	Unfiltered	0	Wheat flour	Control	0	NA	0	NA

^*a*^ Label denotes the label used to identify the sample; air denotes aerobic environmental samples were fermented in air; day denotes the day the starter was sampled; source, source of the sample; chlorine is the indicator of chlorine presence.

^*b*^ *intI1*, relative gene abundance of *intI1* detected; NA, not available; PPM, parts per million, chlorine concentration; PPMf, parts per million.

We obtained 1,160,000 pairs of forward and reverse *16S rRNA* amplicon reads with an average sequencing depth per sample of 41,642.9 paired reads. Sequences were processed using the default parameters of *DADA2* v.1.20 (https://benjjneb.github.io/dada2/tutorial.html) (7) as implemented in *R* v.4.11. Using these parameters, with forward reads cut at 240 bp and reverse reads at 225 bp, we retained 980,261 reads (86%) of the total paired reads post-quality check. Post-alignment, we were able to identify 150 unique amplicon sequence variants (ASVs). Taxonomic assignment was performed with *DADA2* native taxa identifier and the IDTAXA software on the Bioconductor package, *DECIPHER*. Training the identifiers used the SILVA v.138.1(8) and RDP v.18 (9) as reference databases. We found a total of 150 unique ASVs, with 82 unique genera in which *Latilactobacillus* was found to be the most present (39.5%) throughout our samples. This data set presents how metagenomics can be used to understand environmental factors such as air and water quality on starter maturation.

## Data Availability

16S amplicon sequences reads were uploaded on 21 November 2021 onto the National Center for Biotechnology Information under the BioProject accession number PRJNA784321 and are available for download.
